# Isolated internal iliac artery aneurysm causing rectal necrosis due to compression early after endovascular repair: A case report

**DOI:** 10.1016/j.ijscr.2019.07.016

**Published:** 2019-07-19

**Authors:** Toru Imagami, Satoru Takayama, Taku Hattori, Ryohei Matsui, Hisanori Kani, Akimitsu Tanaka, Satoshi Kurokawa

**Affiliations:** a Department of surgery, Nagoya Tokushukai General Hospital, Kasugai City, Japan; b Department of Cardiology of Heart Center, Nagoya Tokushukai General Hospital, Kasugai City, Japan; c Department of urology, Nagoya Tokushukai General Hospital, Kasugai City, Japan

**Keywords:** IIAA, internal iliac artery aneurysm, CT, computed tomography, Fr, French gage, AVP, Amplatzer Vascular Plug, Isolated internal iliac artery aneurysm, Endovascular repair, Residual aneurysm, Rectal necrosis

## Abstract

**Introduction:**

Recently, endovascular repair has become the first-line treatment for internal iliac artery aneurysm (IIAA). However, rectal necrosis due to the compression of the residual IIAA early after endovascular repair is rare.

**Presentation of case:**

We present a rare case of a huge, isolated left IIAA that severely compressed the rectum and ureter. The patient underwent emergency endovascular repair; however, rectal necrosis occurred 10 days later because the repair failed to shrink the size of the aneurismal sac.

**Discussion:**

We hypothesize that the compression of the residual IIAA caused rectal necrosis. During open surgery, endovascular repair disrupted blood flow within the IIAA, which probably allowed for aneurysm dissection and residual hematoma removal.

**Conclusion:**

Endovascular repair alone could not immediately release compression on the surrounding organs; however, open surgical removal of aneurysms after successful endovascular repair may be a useful option for IIAAs with compression of surrounding organs.

## Highlights

• Many advantages of endovascular repair for aneurysms have been reported.
• The compression by aneurysms was not resolved early after endovascular repair.
• Rectal necrosis caused by residual aneurysms early after endovascular repair is rare.
• Aneurysm reduction and intestinal resection after embolization was a useful method.
• Embolization performed earlier assisted surgical decompression of aneurysms.

## 1 Introduction

Internal iliac artery aneurysm (IIAA) sometimes becomes sufficiently large to spontaneously rupture or to affect nearby organs, such as the bowel or urinary system, through compression or invasion [1,2]. The currently available treatments for IIAA include either ligation, excision, endoaneurysmorrhaphy, embolization, and endoluminal stenting or a combination of these [1]. Recently, endovascular repair has become the gold standard for the treatment of IIAAs [3,4]. However, endovascular repair alone may be inadequate to treat an IIAA and its complications, particularly in cases with residual aneurysm.

Herein, we present the case of a large, isolated IIAA complicated by bowel and bilateral ureteral obstructions. Although we treated the IIAA using endovascular repair, residual aneurysm compressed the rectum, resulting in necrotic perforation in the rectum early during the postoperative period. Colonic ischemia is known to occur after interruption of internal iliac artery at 1.12%–2.5% [5,6]; however, rectal necrosis caused by the compression of the isolated IIAA early after endovascular repair is rare. Furthermore, surgical removal of residual aneurysms after successful embolization has been poorly documented. The findings presented below will help improve the outcomes of IIAA treatment. This work has been reported in line with the SCARE criteria [7].

## 2 Case presentation

**Fig. 1:**
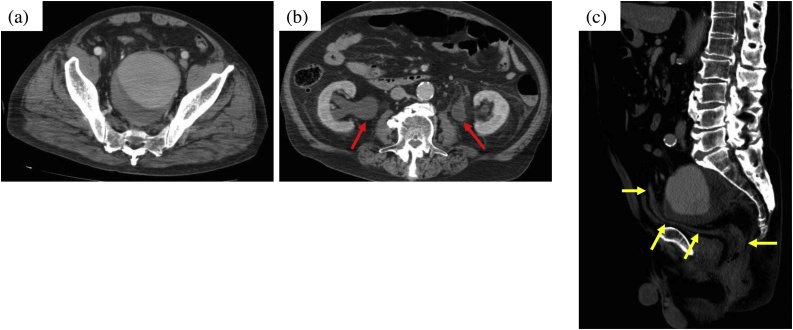
Initial contrast-enhanced CT findings. (a) An isolated internal iliac artery aneurysm (IIAA; maximum diameter, 90 mm) occupying the pelvic cavity. (b) Bilateral hydronephrosis is observed (red arrows), suggesting ureteral obstruction due to the IIAA. (c) The rectum is compressed by the aneurysm, resulting in a narrowing (yellow arrows indicate rectum).

**Fig. 2:**
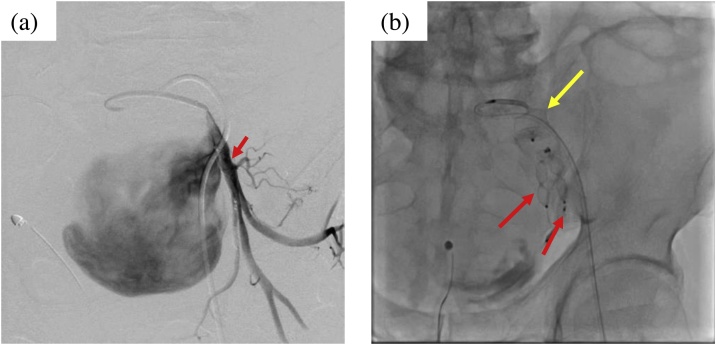
Findings of endovascular repair. (a) Selective angiography of the left internal iliac artery. Distal to the aneurysm, the outflow vessel is slightly bifurcated (red arrow). (b) Embolization with AVP was performed proximal to the internal iliac artery (yellow arrow indicates inflow vessel) and distal to bifurcation of the outflow vessel (red arrows).

A 76-year-old man was referred to a local hospital with a 2-week history of dysuria and constipation. He had a medical history of hypertension and atrial fibrillation, for which he had been prescribed antihypertensives and anticoagulants. When the initial abdominal computed tomography (CT) revealed a large IIAA, the patient was transferred to our hospital for emergency treatment. At our hospital, the biochemical test indicated renal dysfunction (urea nitrogen 60.4 mg/dL, creatinine 3.41 mg/dL and glomerular filtration rate 15 ml/min/1.73m [2]). Contrast-enhanced CT confirmed the presence of a large and isolated left IIAA measuring 90 mm in diameter (Fig. 1). CT images revealed bilateral hydronephrosis along with the compression of the rectum by the IIAA (Fig. 2). However, there was no evidence of intestinal perforation. We therefore established a diagnosis of an isolated, left IIAA with secondary rectal obstruction and bilateral ureteral obstruction.

We anticipated that open surgery would be difficult as the IIAA was large and compressed surrounding organs. We therefore proceeded with endovascular repair. Owing to the significant tortuosity of the right external iliac artery, left femoral artery approach was preferred. Subsequently, emergency endovascular repair was performed after obtaining informed consent from the patient. The left femoral artery was cannulated using a 6-French gage (Fr) sheath catheter. A 4-Fr catheter was used to select the left internal iliac artery and two outflow vessels. As there was no evidence of rupture, the cardiologist deployed a 10-mm Amplatzer Vascular Plug (AVP) II in each outflow vessel and a 16-mm AVP II in the inflow vessel. Hemodynamic stability was maintained throughout the procedure.

**Fig. 3:**
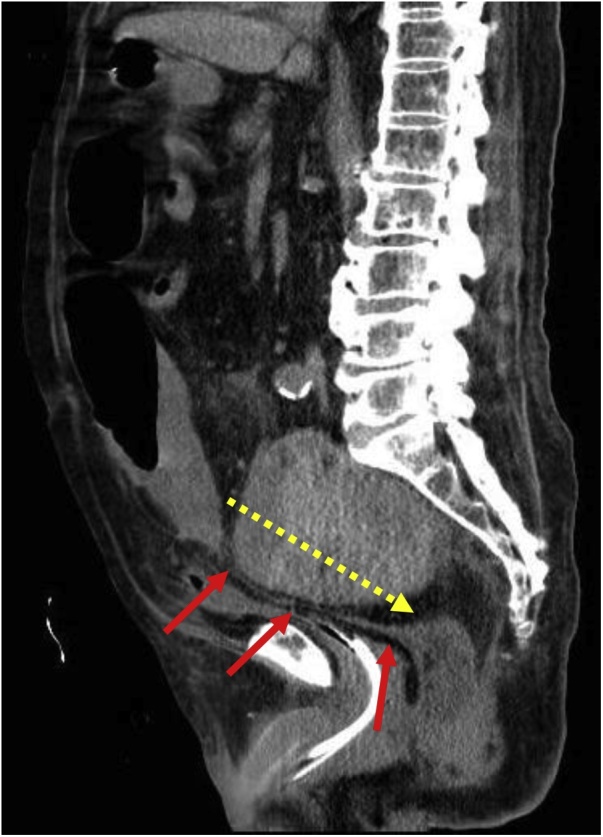
CT findings 10 days after endovascular repair. The rectum is more severely compressed by the aneurysm than before endovascular repair (red arrows). The aneurysm wall was dissected as indicated during open surgery (yellow dotted line).

After successful completion of the endovascular repair, the patient underwent continuous renal replacement therapy. After 3 days, a urologist inserted a ureteral stent into the right ureter; however, he was not able to rescue the left ureter as the deformation was highly severe. The patient also had persistent abdominal distention and limited defecation. Ten days after the endovascular repair, he suddenly developed severe abdominal pain. Abdominal CT at this point revealed intestinal perforation. Emergency open surgery was recommended, and after obtaining written informed consent from the patient and his family, emergency surgery was performed by median laparotomy under general anesthesia. This revealed necrosis and perforation of the anterior wall of the rectum. Compression caused by the aneurysm, which was firmly adherent to the posterior wall of the rectum, had severely damaged the mesorectum. Therefore, the surgeons decided to resect the lesion; however, owing to strong adhesion between the aneurysm and the rectum, incising through the IIAA wall was deemed necessary (Fig. 3). Although a large amount of hematoma was discharged, no active bleeding was observed. The maximal portion of the hematoma was removed. After resection of the affected part of the rectum, a colostomy was formed. Thus, we concluded that the rectum was heavily compressed by the IIAA in the pelvis, resulting in rectal necrosis.

**Fig. 4:**
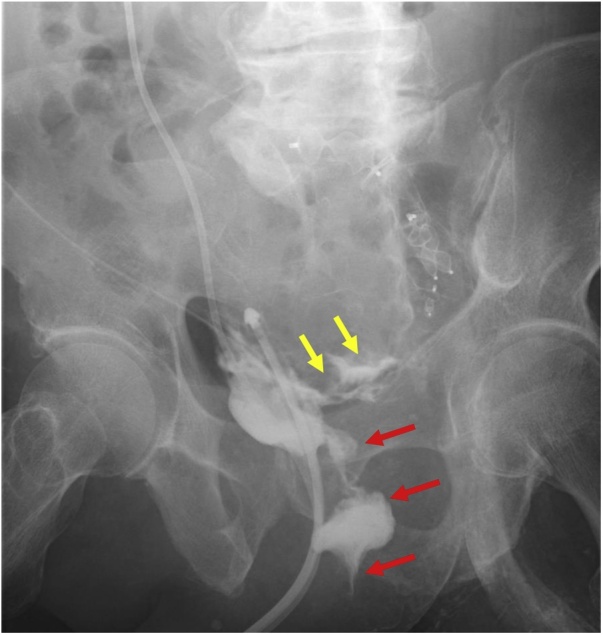
Urethrography performed 18 days after the open surgery. The contrast agent injected from the right ureteral catheter is discharged from the rectum (red arrows). The contrast agent can also be seen leaking into the residual aneurysm (yellow arrows).

Renal replacement therapy proved unnecessary 5 days after the open surgery. Although a vesicorectal fistula developed 18 days after the open surgery (Fig. 4), there was no sign of infection, and the fistula was left untreated. Renal function gradually improved and normalized 4 weeks after endovascular repair. After enough rehabilitation, the patient was discharged 3.5 months following endovascular repair. The patient has been follow-up as an outpatient and has been living well 6 months after endovascular repair.

## 3 Discussion

Most IIAAs are associated with abdominal aortic aneurysms, making isolated IIAAs relatively rare [8]. One review indicated that the typical symptoms of IIAAs include abdominal pain (31.7%), urinary symptoms or renal failure (28.3%), and rectal bleeding or constipation (8.3%) [1]. In the present case, although the size of the aneurysm was extremely large (diameter, 90 mm), the complaints were typical.

Surgical intervention has been universally recommended for IIAAs exceeding 30 mm in diameter because of the possibility of spontaneous rupture [3,9]. In the United States, during 2000–2011, endovascular repair was performed for 65% of patients with IIAAs [10]. Although many advantages of endovascular repair have been reported, this approach cannot be used in every case. We believe that the case presented above will play an important role in determining future treatment strategies for IIAAs.

In this case, we considered initial open surgical repair to be technically challenging and instead preferred endovascular repair. AVP is a versatile embolization agent that allows the operator to treat various conditions including highly challenging vascular lesions [11]. Despite the difficulty in performing endovascular repair in this case, the inflow and outflow vessels were successfully embolized. However, the residual aneurysm that persisted after embolization caused rectal necrosis 10 days after the endovascular repair. These findings demonstrate that the rectum was not relieved from compression by the aneurysm immediately after embolization. In a previous study of endovascular repair for isolated IIAAs, aneurysmal diameter decreased in 59.4% of cases but remained unchanged in 37.5% of cases [12]. This seems to be the greatest disadvantage of endovascular repair for IIAAs, especially in cases involving large aneurysms that compress adjacent organs.

Previous studies have suggested that endovascular repair does not result in rapid aneurysm shrinkage [13,14]. In another report of a case treated with open surgery, the symptoms of compression were considered to be a limitation for performing endovascular repair [15]. However, the rate of mortality associated with use of open surgery ranges from 7% to 11% [1,16]. For the control of intraoperative bleeding, one study reported the use of a hybrid approach that combined proximal open ligation with distal endovascular coil embolization [16]. Thus, we elected to perform a two-stage, hybrid surgery for the open surgical removal of the aneurysm after complete endovascular embolization.

The intraoperative findings in this case revealed that compression of the aneurysm caused the mesorectum to lose its structure; moreover, the aneurysm and the rectum were firmly adherent to each other. We therefore had to remove the aneurysm wall and residual hematoma. This procedure was successfully completed as endovascular repair had disrupted blood flow within the aneurysm. In addition to our case, even in the case of ruptured IIAA to the rectum reported by Kato et al. [8], an open surgery combined with endovascular repair was considered useful.

The limitation of this study is that it is a single-case report. Besides that reported in this case, it has also been reported that an IIAA after endovascular repair can form a fistula within the sigmoid colon [13,17]. Tanase et al. reported that residual aneurysms that do not rupture may cause complications via the pulsatile simulation of nearby organs [13]. There are no guidelines to treat huge size of IIAAs including IIAAs affecting the surrounding organs. Management to prevent the complications of residual aneurysms after endovascular repair is required.

## 4 Conclusion

We presented a case in which compression by a residual IIAA caused rectal necrosis 10 days after endovascular repair. Endovascular repair alone could not immediately release compression on the surrounding organs. However, open surgical removal of aneurysms after successful endovascular repair is considered a useful option for IIAAs with compression of surrounding organs. Further accumulation of cases, both successful and complicated, will be our best attempt for improving the treatment for huge IIAAs.

## Sources of funding

This study did not receive any specific grant from funding agencies in the pubic, commercial, or not-for-profit sectors.

## Ethical approval

This study was approved by the ethics committee in Nagoya Tokushukai General Hospital.

## Consent

Written informed consent was obtained from patient for publication of this case report.

## Author contribution

TI, TH and RM performed open surgery for rectal necrosis. AT performed endovascular repair. SK was responsible for urological examination. TI drafted the manuscript. ST and HK participated in the correction of the manuscript. All authors approved the final manuscript.

## Registration of research studies

No research study involved in this case report. Not applicable.

## Guarantor

Toru Imagami

## Provenance and peer review

Not commissioned, externally peer-reviewed.

## Declaration of Competing Interest

The authors have no conflict of interest to declare.
